# Increase of HO-1 Expression in Critically Ill COVID-19 Patients Is Associated with Poor Prognosis and Outcome

**DOI:** 10.3390/antiox11071300

**Published:** 2022-06-29

**Authors:** Maria G. Detsika, Ioanna Nikitopoulou, Dimitris Veroutis, Alice G. Vassiliou, Edison Jahaj, Stamatis Tsipilis, Nikolaos Athanassiou, Hariklia Gakiopoulou, Vassilis G. Gorgoulis, Ioanna Dimopoulou, Stylianos E. Orfanos, Anastasia Kotanidou

**Affiliations:** 1 1st Department of Critical Care Medicine & Pulmonary Services, GP Livanos and M Simou Laboratories, Evangelismos Hospital, National and Kapodistrian University of Athens, 10675 Athens, Greece; mdetsika@med.uoa.gr (M.G.D.); joannaniki@gmail.com (I.N.); alvass75@gmail.com (A.G.V.); edison.jahaj@gmail.com (E.J.); stamostsipil@gmail.com (S.T.); nikolaosathanasiou14@gmail.com (N.A.); idimo@otenet.gr (I.D.); sorfanos@med.uoa.gr (S.E.O.); 2 Molecular Carcinogenesis Group, Department of Histology and Embryology, Medical School, National and Kapodistrian University of Athens, 10675 Athens, Greece; dimitrisveroutis1@gmail.com (D.V.); vgorg@med.uoa.gr (V.G.G.); 3 First Department of Pathology, Medical School, National and Kapodistrian University of Athens, 11527 Athens, Greece; charagak28@gmail.com; 4 Biomedical Research Foundation, Academy of Athens, 10675 Athens, Greece; 5 Faculty Institute for Cancer Sciences, Manchester Academic Health Sciences Centre, University of Manchester, Manchester M20 4GJ, UK; 6 Center for New Biotechnologies and Precision Medicine, Medical School, National and Kapodistrian University of Athens, 10675 Athens, Greece; 7 Faculty of Health and Medical Sciences, University of Surrey, Surrey GU2 7YH, UK

**Keywords:** heme-oxygenase (HO-1), COVID-19, immune response

## Abstract

Heme-oxygenase (HO)-1 is a cytoprotective enzyme with strong antioxidant and anti-apoptotic properties and previous reports have also emphasized the antiviral properties of HO-1, either directly or via induction of interferons. To investigate the potential role of HO-1 in patients with coronavirus disease 2019 (COVID-19), the present study assessed changes in HO-1 expression in whole blood and tissue samples. Upregulation of HO-1 protein was observed in lung, liver, and skin tissue independently of severe acute respiratory syndrome coronavirus 2 (SARS-CoV-2) presence. A significant increase of blood HO-1 mRNA levels was observed in critically ill COVID-19 patients compared to those in severe COVID-19 patients and healthy controls. This increase was accompanied by significantly elevated levels of serum ferritin and bilirubin in critically ill compared to patients with severe disease. Further grouping of patients in survivors and non-survivors revealed a significant increase of blood HO-1 mRNA levels in the later. Receiver operating characteristic (ROC) analysis for prediction of ICU admission and mortality yielded an AUC of 0.705 (*p* = 0.016) and 0.789 (*p* = 0.007) respectively indicating that HO-1 increase is associated with poor COVID-19 progression and outcome. The increase in HO-1 expression observed in critically ill COVID-19 patients could serve as a mechanism to counteract increased heme levels driving coagulation and thrombosis or as an induced protective mechanism.

## 1. Introduction

Heme-oxygenase (HO)-1 is a cytoprotective enzyme with strong antioxidant and anti-apoptotic properties attributed to its enzymatic activity, which involves the degradation of heme to biliverdin with simultaneous release of carbon monoxide (CO) and ferrous iron. Heme-oxygenase has a strong anti-inflammatory potential by modulating production of interleukins (IL) [1].

The strong anti-inflammatory characteristic of HO-1 has been confirmed in the condition of HO-1 depletion, both in humans and mice. Lack of HO-1 has been shown to result in hyperinflammation with a significant increase in various factors such as IL-1b, IL-6, interferon (IFN)-g, and tumour necrosis factor-a (TNF-a) both in humans and animals [2,3]. Furthermore, mice lacking HO-1 typically show increased sensitivity in sepsis models induced by endotoxins such as lipopolysaccharide (LPS) [4,5]. The same study also reported an induction of HO-1 mRNA levels following LPS administration [5]. Increased HO-1 mRNA levels have also been reported for critically ill patients irrespective of the cause [6].

HO-1 has also been reported to possess antiviral properties, both through its by-products and directly. A previous study reported that activation of HO-1 and CO release inhibited Enterovirus 71 infection by suppressing formation of reactive oxygen species (ROS) [7]. Furthermore, HO-1 was reported to directly interact with interferon regulatory factor 3 (IRF3) thus triggering INFα/β responses and eliminating influenza virus replication in-vitro [8]. The HO-1 expression has also been reported in a cohort of patients with severe disease from Dengue virus showing a strong suppression of expression with disease severity [9] while expression patterns have also been identified in human immunodeficiency (HIV) patients, hepatitis B (HBV) and hepatitis C (HCV) patients [10]. Specifically, a suppression of HO-1 expression was shown in HIV patients with high viral loads [10], while the opposite was observed in patients with HCV suggesting a variable role of HO-1 depending on the type of viral infection and/or type of virus.

Given the strong anti-inflammatory and antiviral potential of HO-1, therapeutic strategies against severe acute respiratory syndrome coronavirus 2 (SARS-CoV-2) have been proposed by various experts [11,12]. However, few studies have yet investigated the profile of HO-1 expression in coronavirus disease 2019 (COVID-19) patients in either tissue or blood level. The current study assessed HO-1 expression levels in blood (mRNA) and tissues (protein) of COVID-19 patients and correlated these levels with disease outcomes.

## 2. Materials and Methods

### 2.1. Patients

A written informed consent was obtained from all patients. Ethical approval was obtained from the Ethics committee of Evangelismos Hospital. All patients had a positive polymerase chain reaction (PCR) test for SARS-CoV-2 performed on a nasopharyngeal sample. A total of 47 patients were recruited. Patients were grouped into those with severe disease (*n* = 27) and critically ill (*n* = 20). Patients were further grouped into survivors (*n* = 38) and non-survivors (*n* = 9). Healthy volunteers with a mean age of 38 years and equal numbers of female and male individuals (*n* = 5) were used as controls (*n* = 10). Blood samples were obtained on admission for all patients and analysed on the same day. Patient demographic and clinical data are shown in Table 1. Formalin-fixed and paraffin-embedded lung, kidney, liver, heart, and skin tissue samples were obtained at autopsy from two non-survivors from the critical patient arm of the study.

### 2.2. Histopathology and Immunohistochemistry

Tissue sections were heat-treated in citric acid buffer and then incubated with 2% H_2_O_2_ to inactivate the endogenous peroxidase. Following blocking with 3% normal goat serum (NGS) for 1 h, sections were incubated with anti-HO-1 monoclonal antibody (cat no: ADI-SPA-895, Enzo Life Sciences, Farmingdale, NY 11735, USA) at a 1:100 dilution overnight at 4 °C. After incubation with an HRP-conjugated secondary antibody, binding was detected using Vectastain ABC Kit (Vector Laboratories, Burlingame, CA, USA) according to manufacturer’s instructions. The 3,3-diaminobenzidine was used as chromogen and slides were counterstained with haematoxylin and observed under an Olympus (BX50F4) microscope (Centre Valley, PA 18034, USA). For SARS-CoV-2 tissue staining with an anti-SARSCoV-2 (G2) monoclonal antibody [13] was performed at a 1:300 dilution as previously described [13]. Routine procedures for Prussian blue (detection of hemosiderin, a ferritin complex) staining were carried out.

### 2.3. RNA Extraction, Reverse Transcription and Real-Time PCR

For RNA extraction blood samples were collected in Tempus Blood RNA Tubes (Applied Biosystems, Foster City, CA, USA) and stored at −80 °C. RNA was extracted according to the manufacturer’s instructions. The RNA concentration was determined for each sample prior to reverse transcription using NanoDrop One (ThermoScientific, Whaltham, MA 02451, USA). Reverse transcription reactions were performed using 4 µL of the 5× FastGene^®^ Scriptase II ReadyMix (Nippon Genetics, Duren, Germany) and 100 ng of RNA for each reaction. Reactions were carried out in a CFX90 cycler (BioRad, Hercules, CA, USA) at the following conditions: 25 °C for 10 min, 42 °C for 60 min and 85 °C for 5 min. Real time PCR reactions were carried out in a CFX90 cycler. Each reaction consisted of 1 μL primer-probe assay mix (IDT, Coralville, IA, USA), 10 μL Luna Master Mix (Biolabs, Waltham, MA, USA) and 1 μL cDNA. The GAPDH was used as a housekeeping gene for data normalization. The sequences for the Real time PCR primers and probes for HO-1 mRNA were as follows: forward primer: 5′-GTT CCT CAT GAA CTC AGC ATT-3′, reverse primer: 5′-GAG CCA GCA CGA ACG AG-3′, probe: 56-FAM/AGC ATG CCC /ZEN/CAG GAT TTG TCA GA/3IABkFQ. Reactions were carried out in triplicate and results were analysed by the ΔΔCT method

### 2.4. Statistical Analysis

Results are reported as absolute numbers, medians, or means and standard deviations, as appropriate. Statistical analysis was performed using the GraphPad Prism 8.0 software for Windows. Data were tested for normality using the Shapiro–Wilks test. Unpaired *t*-test or Mann–Whitney U was used in the case of data displaying normality or not respectively. One-way Anova was used for analysis of three groups and the Fisher’s least significant difference (LSD) test was used for post-hoc analysis. Receiver operating characteristic (ROC) analysis was performed using ICU admission, or survival as the classification variable and HO-1 mRNA levels on admission as prognostic variables. *p* < 0.05 was considered statistically significant.

## 3. Results

### 3.1. Tissue HO-1 Induction Is Independent from SARS-CoV-2 Presence

**Figure 1:**
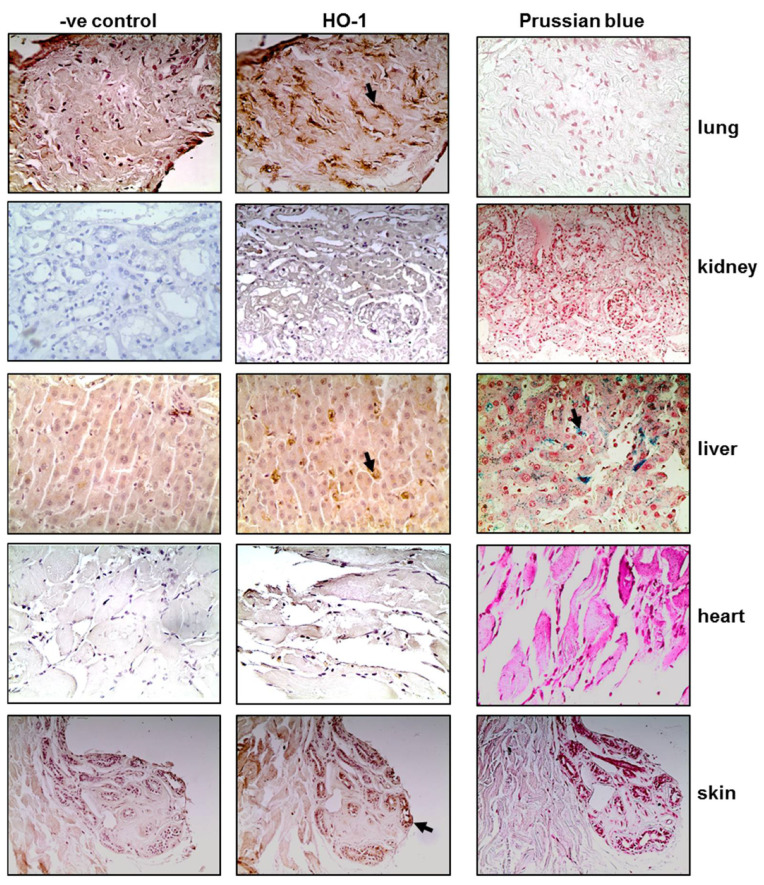
HO-1 upregulation and hemosiderin deposition in tissues of critical COVID-19 patients. Representative images from lung, kidney, liver, heart, and skin tissue sections obtained from critical COVID-19 patients autopsy samples and stained for HO-1 protein (and their respective controls) and Prussian blue. Magnification at ×400.

**Figure 2:**
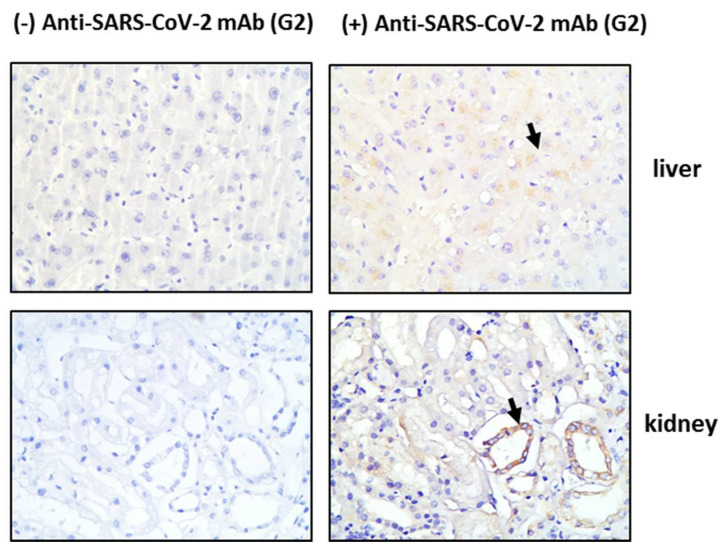
SARS-CoV-2 detection in tissue tissues from critical COVID-19 patients. Post-mortem tissue samples were obtained and directly processed for detection of SARS-CoV-2 by immunohistochemistry. Representative images of SARS-CoV-2 (G2 mab) staining in liver and kidney tissue sections and their respective negative controls. Magnification at ×400.

Tissue sections obtained post-mortem from critically ill COVID-19 patients were stained for HO-1 and hemosiderin to assess whether HO-1 induction occurred at the tissue level and whether it was accompanied by Fe deposition. As shown in Figure 1, HO-1 localization was observed in lung, liver, and skin tissue sections. Specifically, HO-1 immunohistochemical expression in the lung was observed in cells lining the cleft-like spaces which are probably endothelial cells, and in cells within the cleft-like spaces most possibly macrophages (point with arrow). In liver, HO-1 immunohistochemical expression was mainly observed in Kupffer cells (arrow) within the liver sinusoids while a weak granular stain was also discerned in the cytoplasm of hepatocytes with a perinuclear localization. In the same locations, Prussian blue histochemistry revealed abundant granular pigments indicative of ferric iron. The HO-1 immunohistochemical expression was also detected in skin tissue. Skin sweat glands with HO-1 expression were observed in the cytoplasm of epithelial cells without Fe deposition. The HO-1 was not detected in kidney or heart tissue sections and these were also negative for Prussian blue staining. To assess whether HO-1 localized in tissues that were positive for presence of SARS-COV-2, tissue sections demonstrating HO-1 induction were further stained for SARS-CoV-2 protein. The SARS-CoV-2 was only detected in the liver and the kidney (Figure 2); SARS-CoV-2 was not detected in any other tissue sample (Figure S1).

### 3.2. HO-1 Induction in Blood Samples of COVID-19 Patients

**Figure 3:**
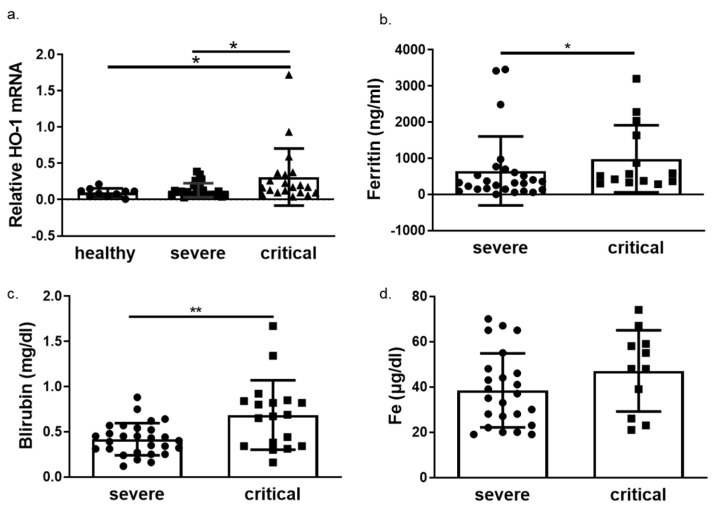
HO-1 is upregulated in critical COVID-19 patients with concomitant increase in ferritin and bilirubin. Whole blood samples were obtained on admission from patients with either severe or critical disease and processed directly. (**a**) HO-1 mRNA upregulation determined in critical patients versus severe and healthy controls from whole blood samples. Increase of ferritin (**b**), bilirubin (**c**) and iron levels (**d**) in critical versus severe COVID-19 patients. Data are expressed as means ± SD. Statistical analysis was performed by one-way ANOVA in three group comparisons and Mann–Whitney U test in two group comparisons. * *p* < 0.05, ** *p* < 0.01. Analysis of variance and post hoc analysis was performed by Uncorrected Fisher’s least significant difference test.

**Figure 4:**
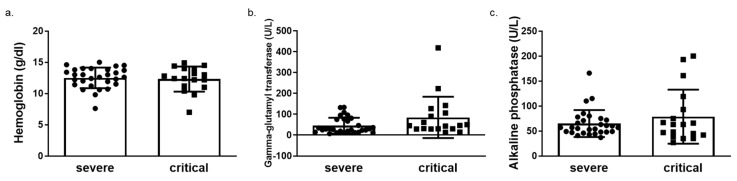
Unaltered levels of haemoglobin, gamma glutamyl transferase (GGT) and alkaline phosphatase (ALP) levels in critical versus severe COVID-19 patients. Levels of (**a**) haemoglobin (**b**) gamma glutamyl transferase (GGT) and (**c**) alkaline phosphatase (ALP) in blood samples obtained on admission from COVID-19 patients by routine in-hospital blood analysis. Data are expressed as means ± SD. Statistical analysis was performed by Mann–Whitney U test. No statistically significant changes were observed.

We next determined HO-1 induction in whole blood samples of COVID-19 patients obtained on admission in either ward or ICU. The HO-1 mRNA levels were significantly elevated in critically ill COVID-19 patient group compared to those in both healthy and severe disease groups (Figure 3a). There was no significant difference in HO-1 mRNA levels between severe disease and healthy control groups (Figure 3a). In addition to the increase in HO-1 mRNA in the critically ill group, there was also an increase in serum ferritin (Figure 3b), bilirubin (Figure 3c) and iron (Fe) levels (Figure 3d). This increase was statistically significant between the critically and severely ill groups for all parameters apart from Fe levels. Furthermore, there was no difference in haemoglobin, gamma-glutamyl transferase (GGT) and alkaline phosphatase (ALP) levels between the critically and severely ill groups (Figure 4).

### 3.3. HO-1 Induction in COVID-19 Patients Is Associated with Disease Progression and Mortality

**Figure 5:**
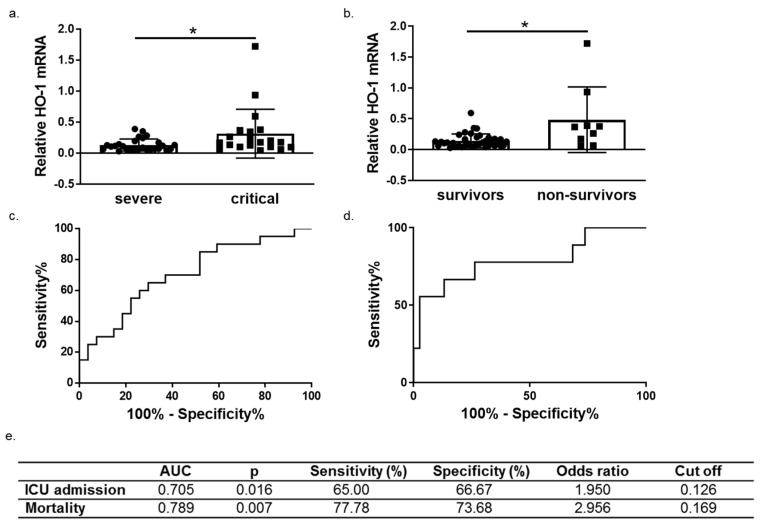
HO-1 mRNA levels are associated with COVID-19 progression and outcome. HO-1 mRNA upregulation in whole blood samples from (**a**) critical versus severe patients (**b**) survivors versus non-survivors. Receiver operating characteristic (ROC) curves of HO-1 mRNA levels for prediction of need for intensive care (**c**) and mortality (**d**). The corresponding areas under the curve (AUC), p, sensitivity and specificity, odd ratio and cut off values are represented in (**e**). Statistical analysis was performed by Mann–Whitney U test. * *p* < 0.05.

We next performed ROC curve analysis to assess whether HO-1 induction predicts COVID-19 outcomes (progression and mortality). To this end, patients were further grouped into survivors (*n* = 38) and non-survivors (*n* = 9). A significant increase of HO-1 whole blood mRNA levels was also observed in non-survivors versus survivors (Figure 5b). The ROC curves were generated for prediction of ICU admission and mortality and revealed an AUC of 0.705 (95% 0.5545–0.8566, *p* = 0.016) and 0.789 (95% 0.5977–0.9813, *p* = 0.015), respectively (Figure 5c,d).

## 4. Discussion

Severe and critical COVID-19 infection largely exhibits characteristics of a multi-inflammatory condition culminating in viral sepsis. The highly pro-inflammatory profile of these patients could be counteracted by the induction of anti-inflammatory molecules such as HO-1. Our results show significantly elevated whole blood HO-1 mRNA levels in critically ill patients indicating that HO-1 induction could be acting as a protective mechanism against the inflammation. The HO-1 has previously been shown to reduce IL-6 and CXCL1 levels in mouse models of ischemia/reperfusion (IRI) models of renal and lung injury [14,15]. To achieve HO-1 induction in these models, hemin was used as a natural HO-1 inducer and was found to reduce IRI cytokine storm. The HO-1 induction in COVID-19 patients could therefore potentially add to reduce the cytokine storm observed. The same studies reported a reduction of lung tissue injury upon hemin induced HO-1 upregulation. 

The HO-1 also has an established antiviral effect. Previous studies have reported upregulation of HO-1 by cobalt protoporphyrin which eliminated Zika virus (ZIKV) replication in vitro in HEK-293A cells expressing a ZIKV replicon [16]. Another study described the HO-1 dependent inhibition of Ebola viral replication following HO-1 induction by cobalt protoporphyrin (CoPP) [17]. Furthermore, hemin treatment of monocytes and T cells previously infected with HIV pleiotropic strains was shown to ameliorate viral infection in vitro while tin protoporphyrin, an established inhibitor of HO-1 activity was shown to attenuate HIV infection indicating the antiviral effect of the enzymatic role of HO-1 [18]. A similar study conducted for SARS-CoV-2 recently reported no effect of HO-1 upregulation on SARS-CoV-2 replication in vitro. Specifically, the study utilized monkey kidney and human lung epithelial cell lines, infected with SARS-CoV-2 and assessed the antiviral effect of HO-1 by induction through hemin at 48 and 72 h post infection with no significant reduction in viral replication [19] despite the augmented HO-1 expression.

In our study, HO-1 was detected in lung, liver, and skin tissue sections of critically ill patients. The detection of HO-1 in tissue samples was independent from SARS-CoV-2 presence as HO-1 presence was confirmed in lung and skin tissue sections that were negative for SARS-COV-2, while absence of HO-1 was observed in renal tissue that was positive for SARS-COV-2. This observation indicates that the induction of HO-1 targets COVID-19 independent of a direct antiviral effect possibly because of the systemic inflammatory response triggered during the infection. A limitation of our study regarding staining of tissues from COVID-19 patients is the lack of healthy controls as well as the limited number of patients included in the study (*n* = 2). Instead, we have used a negative control of the same tissue sample. Therefore, although HO-1 was detected in specific tissue samples we could not assess the degree of protein expression. Another study has also described the detection of HO-1 in skin tissue samples from COVID-19 patients [20]. However, the study included patients with mild symptoms and the specimens were obtained from specific skin lesions and reported downregulation of HO-1 protein expression when compared to healthy controls. Further analysis in samples from critically ill patients is needed to confirm our results. 

The increase of HO-1 whole blood mRNA levels observed in critical COVID-19 patients was coupled to increased Fe, albeit not significantly, and a significant increase in bilirubin (Figure 3). Whether the increase in Fe and bilirubin levels could be attributed to the HO-1 induction (which releases heme-bound iron and generates bilirubin) is difficult to assess. However, the fact that GGT and ALT levels were no different between severe and critically ill patients (Figure 4) indicates that the increase in bilirubin was not due to liver damage. The elevated levels of Fe observed in these patients, however, could be another mechanism for HO-1 induction. Specifically, Fe is needed for heme synthesis, which is the natural inducer of HO-1. The Fe is usually bound by ferritin, the levels of which were increased in tandem with HO-1 mRNA increase (Figure 3). Any unbound Fe may therefore be captured for heme synthesis leading to HO-1 induction. However, apart from liver tissue, the induction of HO-1 in tissue levels was not always associated with Fe deposition. 

The HO-1 mRNA levels were shown to be significantly elevated in non-survivors and ROC analysis showed a high AUC value indicating that this increase of HO-1 expression is associated with mortality. Whether this association of HO-1 with mortality is due to the upregulation of HO-1 as a protective mechanism or whether the overexpression of HO-1 may intensify a ‘lethal’ effect remains to be unravelled. Specifically, it has been shown that apart from its beneficial properties, HO-1 overexpression may have detrimental effect both at tissue and circulation level and this is mainly attributed to the release of heme-derived catalytically active iron which has been shown to promote a pro-oxidant environment [21,22]. In our study, Fe blood levels were increased in critically ill patients but not significantly. Furthermore, circulating iron levels reflect transferrin bound iron and not heme derived iron. Moreover, iron deposition at tissue levels was only observed in the liver of critically ill patients indicating that the upregulation of HO-1 remains within the ‘non-toxic threshold’ of expression. Taken together, the observations above, indicate that the association of HO-1 upregulation with mortality of COVID-19 patients is probably due to its induction as a protective mechanism rather than a toxic epiphenomenon of the course of COVID-19 viral infection.

This study showed a strong association of increased HO-1 expression and disease progression as well as outcome, specifically, mortality. The high AUC values shown for the need for ICU treatment and mortality allow for a suggestion of the potential use of HO-1 as a biomarker for COVID-19 disease progression and outcome. However, considering the limited size of the study population, HO-1 may be proposed as a strong indicator rather that a biomarker of COVID-19 progression and mortality. Validation of HO-1 expression levels in larger COVID-19 patient cohorts is therefore necessary to confirm the potential use of HO-1 as a prognostic tool for COVID-19 management and to compare its performance with other established parameters used for COVID-19 disease progression, such as C-reactive protein (CRP) and the neutrophil to lymphocyte ratio (NLR). Another study previously reported elevated levels of HO-1 in COVID-19 patients with low oxygen levels (SpO2 ≤ 95%) [23], measured by a standard ELISA method accompanied by increased heme levels. However, the study was also performed on a limited number of patients and stressed the need for further investigation in larger cohorts. Finally, one study reported the use of HO-1 as a marker for disease progression in a patient with exacerbation of idiopathic pulmonary fibrosis (IPF) following SARS-CoV-2 infection and COVID-19, and they found that serum HO-1 which reflected M2 macrophage activation allowed monitoring of the disease progression [24]. However, the study was focused specifically on patients with exacerbation of IPF following COVID-19 and did not suggest HO-1 as a marker for COVID-19 progression in general.

## 5. Conclusions

This study describes the expression pattern of HO-1 in COVID-19 patients, both at the tissue level and the blood mRNA level. The increase of HO-1 in critically ill and disease COVID-19 patients indicates that its induction serves as a protective mechanism. Further studies are needed to determine the underlying mechanisms for HO-1 induction in COVID-19 to directly assess its potential as a therapeutic strategy against COVID-19.

## Figures and Tables

**Table 1:** Patient clinical and demographic data.

	Severe COVID-19	Critical COVID-19	*p* Value
Age (years)	60.26 ± 15.96	66.65 ± 11.45	0.149
Male	12 (44.44%)	15 (75.00%)	0.111
Comorbidities	19 (70.37%)	15 (75.00%)	0.940
Symptoms			
paO2/FIO2	318.80 ± 63.62	137.90 ± 79.23	<0.0001
Days of illness before admission	5.65± 1.62	5.77 ± 2.98	0.852
Laboratory baseline			
White blood cells (cells/µL)	6664.00 ± 3841	13897 ± 1231	<0.0001
Neutrophils (cells/µL)	68.34 ± 16.62	79.80 ± 13.01	0.012
Lymphocytes (cells/µL)	27.58 ± 19.17	12.02 ± 13.30	0.001
Platelets (cells/µL)	207333 ± 88056	271737 ± 139869	0.100
C-reactive protein (mg/L)	7.05 ± 7.57	13.67 ± 11.52	0.025
Troponin (ng/mL)	37.58 ± 109.70	311.80 ± 845.90	0.114
Urea (mg/dL)	34.89 ± 24.78	84.16 ± 94.24	<0.001
Creatinine (mg/dL)	0.82 ± 0.19	1.10 ± 0.88	0.368
Aspartate aminotransferase (U/L)	43.33 ± 37.50	150.70 ± 419.20	0.237
Alanine transaminase (U/L)	34.76 ± 22.94	62.17 ± 74.60	0.002
Gamma-Glutamyltransferase (U/L)	46.37 ± 39.22	83.26 ± 96.73	0.062
Lactate Dehydrogenase (U/L)	306.8 ± 128.9	535.6 ± 552.1	0.021
Albumin (g/dL)	3.846 ± 0.409	3.210 ± 0.506	<0.0001
Days of hospital stay	8.00 ± 4.019	18.80 ± 14.10	<0.0001
Survival	25 (92.59%)	12 (60.00%)	0.026

Data are presented as mean ± SD or absolute number (percentage of group).

## Data Availability

The data is contained within the manuscript and the Supplementary Material.

## Supplementary Materials

The following supporting information can be downloaded at https://www.mdpi.com/article/10.3390/antiox11071300/s1. Figure S1: SARS-COV-2 absence in tissue samples from critical COVID-19 patients.

## References

[1] Maines M.D. (1997). The heme oxygenase system: A regulator of second messenger gases. Annu. Rev. Pharmacol. Toxicol..

[2] Grochot-Przeczek A., Dulak J., Jozkowicz A. (2012). Haem oxygenase-1: Non-canonical roles in physiology and pathology. Clin. Sci..

[3] Poss K.D., Tonegawa S. (1997). Heme oxygenase 1 is required for mammalian iron reutilization. Proc. Natl. Acad. Sci. USA.

[4] Espinoza J.A., Gonzalez P.A., Kalergis A.M. (2017). Modulation of Antiviral Immunity by Heme Oxygenase-1. Am. J. Pathol..

[5] Wiesel P., Patel A.P., DiFonzo N., Marria P.B., Sim C.U., Pellacani A., Maemura K., LeBlanc B.W., Marino K., Doerschuk C.M. (2000). Endotoxin-induced mortality is related to increased oxidative stress and end-organ dysfunction, not refractory hypotension, in heme oxygenase-1-deficient mice. Circulation.

[6] Saukkonen K., Lakkisto P., Kaunisto M.A., Varpula M., Voipio-Pulkki L.M., Varpula T., Pettila V., Pulkki K. (2010). Heme oxygenase 1 polymorphisms and plasma concentrations in critically ill patients. Shock.

[7] Tung W.H., Hsieh H.L., Lee I.T., Yang C.M. (2011). Enterovirus 71 induces integrin beta1/EGFR-Rac1-dependent oxidative stress in SK-N-SH cells: Role of HO-1/CO in viral replication. J. Cell. Physiol..

[8] Ma L.L., Zhang P., Wang H.Q., Li Y.F., Hu J., Jiang J.D., Li Y.H. (2019). Heme oxygenase-1 agonist CoPP suppresses influenza virus replication through IRF3-mediated generation of IFN-alpha/beta. Virology.

[9] Wu Y.H., Chen W.C., Tseng C.K., Chen Y.H., Lin C.K., Lee J.C. (2022). Heme oxygenase-1 inhibits DENV-induced endothelial hyperpermeability and serves as a potential target against dengue hemorrhagic fever. FASEB J. Off. Publ. Fed. Am. Soc. Exp. Biol..

[10] Jablonowska E., Wojcik K., Szymanska B., Omulecka A., Cwiklinska H., Piekarska A. (2014). Hepatic HMOX1 expression positively correlates with Bach-1 and miR-122 in patients with HCV mono and HIV/HCV coinfection. PLoS ONE.

[11] Wagener F., Pickkers P., Peterson S.J., Immenschuh S., Abraham N.G. (2020). Targeting the Heme-Heme Oxygenase System to Prevent Severe Complications Following COVID-19 Infections. Antioxidants.

[12] Rossi M., Piagnerelli M., Van Meerhaeghe A., Zouaoui Boudjeltia K. (2020). Heme oxygenase-1 (HO-1) cytoprotective pathway: A potential treatment strategy against coronavirus disease 2019 (COVID-19)-induced cytokine storm syndrome. Med. Hypotheses.

[13] Evangelou K., Veroutis D., Paschalaki K., Foukas P.G., Lagopati N., Dimitriou M., Papaspyropoulos A., Konda B., Hazapis O., Polyzou A. (2022). Pulmonary infection by SARS-CoV-2 induces senescence accompanied by an inflammatory phenotype in severe COVID-19: Possible implications for viral mutagenesis. Eur. Respir. J..

[14] Chok M.K., Ferlicot S., Conti M., Almolki A., Durrbach A., Loric S., Benoit G., Droupy S., Eschwege P. (2009). Renoprotective potency of heme oxygenase-1 induction in rat renal ischemia-reperfusion. Inflamm. Allergy Drug Targets.

[15] Rossi M., Delbauve S., Roumeguere T., Wespes E., Leo O., Flamand V., Le Moine A., Hougardy J.M. (2019). HO-1 mitigates acute kidney injury and subsequent kidney-lung cross-talk. Free. Radic. Res..

[16] El Kalamouni C., Frumence E., Bos S., Turpin J., Nativel B., Harrabi W., Wilkinson D.A., Meilhac O., Gadea G., Despres P. (2018). Subversion of the Heme Oxygenase-1 Antiviral Activity by Zika Virus. Viruses.

[17] Hill-Batorski L., Halfmann P., Neumann G., Kawaoka Y. (2013). The cytoprotective enzyme heme oxygenase-1 suppresses Ebola virus replication. J. Virol..

[18] Devadas K., Dhawan S. (2006). Hemin activation ameliorates HIV-1 infection via heme oxygenase-1 induction. J. Immunol..

[19] Maestro S., Cordoba K.M., Olague C., Argemi J., Avila M.A., Gonzalez-Aseguinolaza G., Smerdou C., Fontanellas A. (2021). Heme oxygenase-1 inducer hemin does not inhibit SARS-CoV-2 virus infection. Biomed. Pharmacother..

[20] Cazzato G., Colagrande A., Cimmino A., Cicco G., Scarcella V.S., Tarantino P., Lospalluti L., Romita P., Foti C., Demarco A. (2021). HMGB1-TIM3-HO1: A New Pathway of Inflammation in Skin of SARS-CoV-2 Patients? A Retrospective Pilot Study. Biomolecules.

[21] Suttner D.M., Dennery P.A. (1999). Reversal of HO-1 related cytoprotection with increased expression is due to reactive iron. FASEB J. Off. Publ. Fed. Am. Soc. Exp. Biol..

[22] Kvam E., Hejmadi V., Ryter S., Pourzand C., Tyrrell R.M. (2000). Heme oxygenase activity causes transient hypersensitivity to oxidative ultraviolet a radiation that depends on release of iron from heme. Free. Radic. Biol. Med..

[23] Su W.L., Lin C.P., Hang H.C., Wu P.S., Cheng C.F., Chao Y.C. (2021). Desaturation and heme elevation during COVID-19 infection: A potential prognostic factor of heme oxygenase-1. J. Microbiol. Immunol. Infect..

[24] Hara Y., Oshima Y., Tagami Y., Aoki A., Fujii H., Izawa A., Seki K., Kanai A., Yabe A., Watanabe K. (2022). Clinical importance of serum heme oxygenase-1 measurement in patients with acute exacerbation of idiopathic pulmonary fibrosis triggered by coronavirus disease 2019. Respir. Med. Case Rep..

