# Supplementary material for: Increase of HO-1 Expression in Critically Ill COVID-19 Patients Is Associated with Poor Prognosis and Outcome
Source: Antioxidants (Basel). 2022 Jun 29;11(7):1300. doi: 10.3390/antiox11071300 (PMC9311906; doi:10.3390/antiox11071300)

antioxidants

MDPI

Supplementary Materials: Increase of HO-1 Expression in Critically Ill COVID-19 Patients Is Associated with Poor Prognosis and Outcome

Increase of HO-1 Expression in Critically Ill COVID-19 Patients Is Associated with Poor Prognosis and Outcome

Maria G. Detsika [1], Ioanna Nikitopoulou [1], Dimitris Veroutis [2], Alice G. Vassiliou [1], Edison Jahaj [1], Stamatis Tsipilis [1], Nikolaos Athanassiou [1], Hariklia Gakiopoulou [3], Vassilis G. Gorgoulis [2,4,5,6,7], Ioanna Dimopoulou [1], Stylianos E. Orfanos [1] and Anastasia Kotanidou [1,*]

[1] 1st Department of Critical Care Medicine & Pulmonary Services, GP Livanos and M Simou Laboratories, Evangelismos Hospital, National and Kapodistrian University of Athens, 10675 Athens, Greece; mdetsika@med.uoa.gr (M.G.D.); joannaniki@gmail.com (I.N.); alvass75@gmail.com (A.G.V.); edison.jahaj@gmail.com (E.J.); stamostsipil@gmail.com (S.T.); nikolaosathanasiou14@gmail.com (N.A.); idimo@otenet.gr (I.D.); sorfanos@med.uoa.gr (S.E.O.)

[2] Molecular Carcinogenesis Group, Department of Histology and Embryology, Medical School, National and Kapodistrian University of Athens, 10675 Athens, Greece; dimitrisveroutis1@gmail.com (D.V.); vgorg@med.uoa.gr (V.G.G.)

[3] First Department of Pathology, Medical School, National and Kapodistrian University of Athens, 11527 Athens, Greece; charagak28@gmail.com

[4] Biomedical Research Foundation, Academy of Athens, 10675 Athens, Greece

[5] Faculty Institute for Cancer Sciences, Manchester Academic Health Sciences Centre, University of Manchester, Manchester M20 4GJ, UK

[6] Center for New Biotechnologies and Precision Medicine, Medical School, National and Kapodistrian University of Athens, 10675 Athens, Greece

[7] Faculty of Health and Medical Sciences, University of Surrey, Surrey GU2 7YH, UK

* Correspondence: akotanid@med.uoa.gr

Supplementary Figure S1. SARS-COV-2 absence in tissue samples from critical COVID-19 patients. Representative images of SARS-CoV-2 (G2 mAb) staining in lung, heart and skin tissue sections which were negative for SARS-COV-2 presence. Magnification at X400.

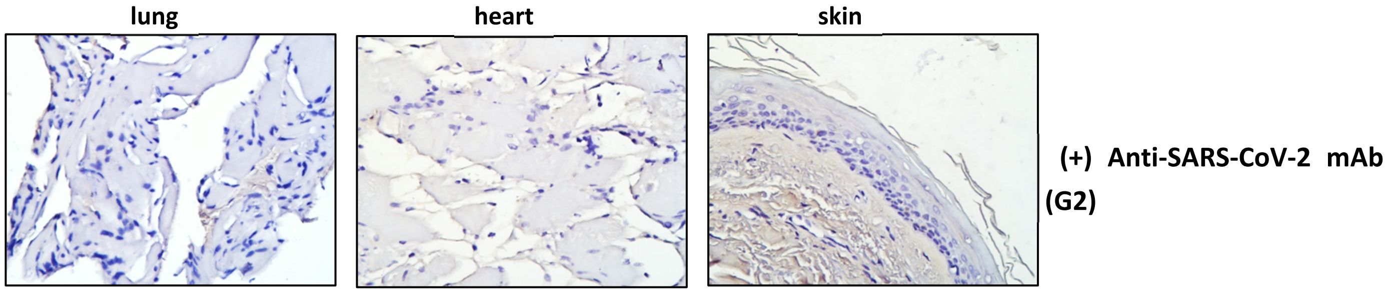

Supplement: Supplementary file 1 [file antioxidants-11-01300-s001.zip › antioxidants-1779225-supplementary.pdf]
